# Safety outcomes of the first Rigi10™ malleable penile prostheses implanted worldwide

**DOI:** 10.1038/s41443-023-00761-x

**Published:** 2023-09-07

**Authors:** Steven K. Wilson, Lexiaochuan Wen, Rafael Carrion, Francois Eid, Aaron Lentz, Bhavik Shah, Orhan Koca, Muhammed A. M. Hammad, Vijayant Govinda Gupta, Mohammad Hamdan

**Affiliations:** 1 Institute for Urologic Excellence, La Quinta, CA USA; 2 Department of Urology, Mayo Clinic, Rochester, MN USA; 3 Department of Urology, University of South Florida, Tampa, FL USA; 4 Advanced Urological Care, Norwell Health, New York, NY USA; 5 Department of Urologic Surgery, Duke University, Durham, NC USA; 6 Advanced Urology, Snellville, GA USA; 7 Department of Urology, Medistate Kavacik Hospital, Istanbul, Turkey; 8 Department of Urology, University of California Irvine Health, Orange, CA USA; 9 Department of Urology, Govinda Medicentre, New Delhi, India; 10 Department of Urology, Royal Hospital, Amman, Jordan

**Keywords:** Erectile dysfunction, Risk factors

## Abstract

Implantation of penile prosthesis is considered when conservative measures fail or are unacceptable to patients’ wishing treatment for erectile dysfunction. In the United States (US), inflatable penile prostheses are more often used than malleable penile prostheses (MPP). Outside the US, the reverse is true because third-party reimbursement is not available, and MPP is considerably cheaper. Two American manufacturers make MPP; presently, a new manufacturer, Rigicon (Ronkonkoma NY), has recently begun to sell its MPP worldwide. Patient information forms submitted to the manufacturer between March 1, 2019, and December 8, 2022, were used to conduct an initial safety study for 605 first-time patients implanted with Rigicon10® by 46 physicians in 15 countries with a mean follow-up of 21.6 months. It has the same configuration of trimmable, paired silicone rods containing a twisted stainless-steel wire for bendability. However, it is available in six widths with hydrophilic coating compared to three widths offered by competitors. Revision or explantation was needed in 6 of 605 patients (0.99%) with half of those being removed for dissatisfaction (0.50%). Two (0.33%) suffered device infection and one (0.16%) required removal for erosion. Kaplan–Meier’s statistical analysis showed three-year implant survival from revision = 99.2%. It demonstrated a comparable safety record with less than 1.00% of patients requiring reoperation.

## Introduction

Erectile dysfunction (ED) is the inability to achieve or maintain an erection suitable for sexual intercourse [1]. Etiologies are generally organic with vasculogenic (arterial and venous pathologies) being the most common [1]. Contributing etiologies include pelvic and prostate surgery, Peyronie’s disease and diabetes [1]. Conservative measures like lifestyle change, oral medications, vacuum devices, and penile injections of intracorporal or intraurethral vasoactive material may help mild to moderate ED [2]. Penile prosthesis implantation is employed when other treatment alternatives fail, cannot be used or if the patient desires [2]. Penile prosthesis implantation aims to obtain an erection that mimics physiological erection with minimal complication and/or mechanical failure [2]. The device also should provide patient satisfaction after the treatment [3]. Nowadays, innovative surgical techniques and enhancements to prostheses by manufacturers have meaningfully decreased implant surgery complications and increased device longevity from revision surgery [4, 5].

The most common penile implants are semi-rigid malleable penile prosthesis (MPP) and multicomponent inflatable penile prosthesis (IPP). In the United States (US), unlike many countries in the world, the inflatable models are reimbursed by government insurance and many private insurers. When we compare inflatable models to malleable ones, the IPP has better patient and partner satisfaction and in the US, despite the higher cost, accounting for 90% of penile implants [6]. In the rest of the world, where third-party reimbursement is less common, the considerably cheaper MPP is utilized more often than IPP. In the US, two domestically manufactured MPPs have been available for decades. Boston Scientific currently makes and sells Tactra® and Coloplast manufactures and markets Genesis®. Rigicon introduced their MPP, the Rigi10®, in 2019. While it has been widely used overseas, the Rigi10® has only been available within the US market starting in 2021. In this multi-center across 15 countries, we aim to evaluate the first reported safety outcomes of the new Rigi10® device.

## Methods

We utilize patient information forms (PIFs) filled out at the time of surgery and mailed back to the manufacturer to obtain our data. Historically, there have been many seminal penile prosthesis studies utilizing PIF data and Institutional Review Board approval has not been necessary due to the anonymity of the patient [7, 8]. Prior to our analysis, all patient identifying information had been removed from the PIF and only the patient’s hospital number and the name of the surgeon remained on the form. Information on the PIF included patient demographics, date of operation, model, size of components, surgical incision location, and reason for reoperation if necessary.

### Features of Rigi10® MPP

**Fig. 1:**
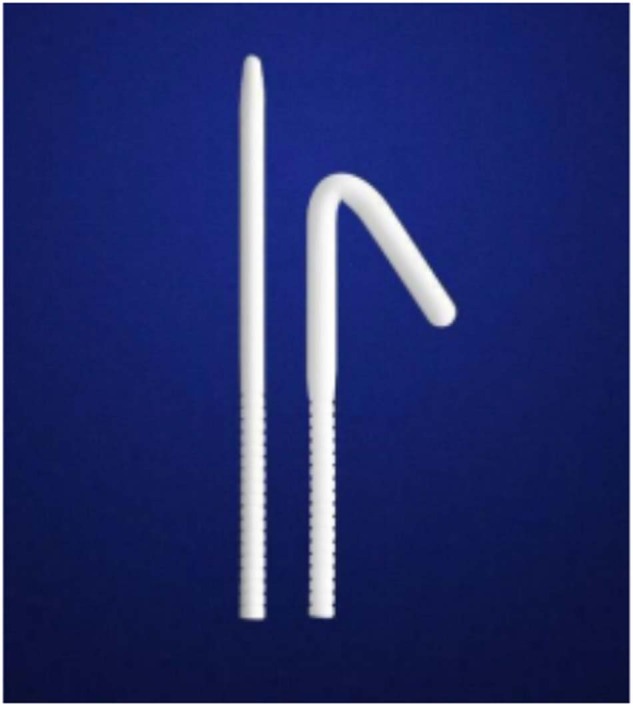
The Rigicon Rigi10® malleable penile prosthesis.

Rigi10® MPP is a sterile, trimmable, and single-use implant that consists of two cylinders available in two lengths and six different widths, plus extender tips (Fig. 1). The efficiency of trimmable cylinders with extenders makes it useful for patients who have varying corporal lengths. Boston Scientific and Coloplast competitive devices are supplied in 9-, 11- and 13-mm widths. Physicians may inadvertently oversize the girth of the cylinder when restricted to only three widths because they wish to give the patient maximum penile girth, which equates to the best penile rigidity. An added feature of the Rigi10® is the introduction of two additional “in between” sizes—10 and 12 mm—making it possible to fit the width of the cylinders more accurately to the individual penis. The cylinders have a stainless-steel wire, which is quite flexible, improving concealability in the patient’s clothing. The rod is covered with Teflon and silicone. The Rigi10™ provides the required rigidity, comfort, and discretion to patients with ED. Since this study uses PIFs of the earliest used devices, almost all the Rigi10® included had a hydrophilic coating meant to retard infection. Although the model presently sold in the US does have a hydrophilic coating, the few earliest US implants that were included in the study did not. The hydrophilic coating was initially omitted on those devices for regulatory reasons.

### Study population

A total of 605 patients with ED of various etiologies who underwent first-time surgery from 15 different countries by 46 urologists between March 1, 2019, and December 8, 2022, were reviewed. The mean patient age was 58.7 ± 4.74 [49–75] years, and the follow-up time was 21.6 ± 8.26 [9–33] months. The data of all patients were analyzed to identify ED etiology and postoperative outcome. These were PIFs from the first patients implanted with the new Rigicon MPP. The PIF accompanies the packaged implant from the factory and is filled out by implanting physicians, hospital personnel in the operating room, or company employees in attendance at the surgery.

Physicians, nurses and vendors are compulsive about filling out these forms and making certain they are mailed back to the manufacturer because a PIF is necessary to activate the lifetime replacement guarantee of the Rigicon device. All patients in the study were first-time implants.

Patients excluded were those with previous penile implantation or augmentation, patients who had contraindications to general anesthesia, known allergies, or sensitivity to product materials as indicated in the device labeling.

### Surgical technique

Three surgical approaches were utilized for the implantation of the Rigicon Rigi10® MPP: infrapubic, penoscrotal, and subcoronal. The erectile tissue of the cavernosa was dilated, measured, and implanted with the same-sized penile prosthesis to fit the patient’s particular anatomy. The width of the prosthesis cylinders was determined by placing two dilators of similar size simultaneously in the corpora distally and checking for a snug fit. The prostheses that were inserted in the patients had diameters of 9, 10, 11, 12, and 13.0 mm. and were either 23 cm for 9 and 10 mm or 25 cm for the larger widths. A 14-mm rod recently became available for the Rigi10®, but it was not employed in this study.

### Statistical analysis

The mean standard deviation values of the cases were used to compare the characteristics of the patients who underwent revision and non-revision surgeries. Kaplan–Meier survival statistics were calculated using SPSS 22.0 (IBM, US) software.

## Results

ED etiologies include diabetes mellitus (*n* = 270; 44.6%), organic ED (but not otherwise classified) (*n* = 199; 32.96%), prostatectomy (*n* = 47; 7.76%), vascular diseases (*n* = 26; 4.29%), Peyronie’s disease (*n* = 26; 4.29%), radical surgery (*n* = 12; 1.98%), other (*n* = 15; 2.48%), spinal cord trauma (*n* = 9; 1.48%), priapism (*n* = 1; 0.16%) (Table 1). These data are not complete because it is regrettable that the original PIF form mistakenly allowed the etiology of ED to be considered organic without further breakdown into vascular, post-surgical, diabetes, etc. This is the same problem we had with the PIF-generated study of the Rigicon Infla10® IPP [9]. The PIF for both prostheses was corrected late in the study.

**Table 1:** Etiology of erectile dysfunction.

Diabetes mellitus	270	44.60%
Organic	199	32.96%
Prostatectomy	47	7.76%
Vascular diseases	26	4.29%
Peyronie’s disease	26	4.29%
Radical surgery	12	1.98%
Other	15	2.48%
Spinal cord trauma	9	1.48%
Priapism	1	0.16%

Of the three most common implant incisions, the most often used was the penoscrotal, which was utilized in 93% of the patients (Table 2). The predominant width of the cylinder used was 11 mm at 32.2% followed by 12 mm at 28.3% (Table 3).

**Table 2:** Surgical incision location.

Penoscrotal	571	92.66%
Subcoronal	23	5.12%
Infrapubic	10	2.00%
Penoscrotal + subcoronal	1	0.22%

**Table 3:** Width of malleable rod.

9 mm	63	10.41%
10 mm	101	16.70%
11 mm	195	32.23%
12 mm	171	28.27%
13 mm	75	12.39%

The paucity of revision operations was remarkable. Penile prosthesis reoperation or revision was required in 6 of 605 (0.99%) followed for a mean of almost 2 years. Of these, 3 patients (0.05%) were dissatisfied with their MPPs quality of erection and concealability and were ultimately transitioned to IPP. There were two infections requiring removal (0.033%) and there was an erosion in one patient (0.016%). There were no mechanical failures requiring reoperation. Also, notably, there were no instances of glandular ischemia or pain (considered a complication of oversizing width and length, respectively, of MPP) [4]. The lengths of the rear tip extenders and the cavernosal measurements (including the proximal and distal length) were cataloged to detect possible differences between the non-revision group and the revision group (Table 4). No detectable differences were noted in the two groups.

**Table 4:** Corporal measurements differences between non-revision group (*n* = 599) and revision group (*n* = 6).

	Non-revision group (left)	Revision group (left)
Rear tip extenders (cm)	0.56 ± 0.17	0.5 ± 0.0
Total intracavernosal measurements (cm)	19.27 ± 2.14	18.66 ± 3.68
Corporeal measurements (proximal) (cm)	11.33 ± 2.91	9.00 ± 00
Corporeal measurements (distal) (cm)	8.76 ± 2.66	10.00 ± 00

	Non-revision group (right)	Revision group (right)
Rear tip extenders (cm)	0.59 ± 0.36	0.5 ± .00
Total intracavernosal measurements (cm)	19.24 ± 2.22	19.66 ± 2.49
Corporeal measurements (proximal) (cm)	11.03 ± 2.78	9.00 ± 0.0
Corporeal measurements (distal) (cm)	8.64 ± 2.58	10.00 ± 00

**Fig. 2:**
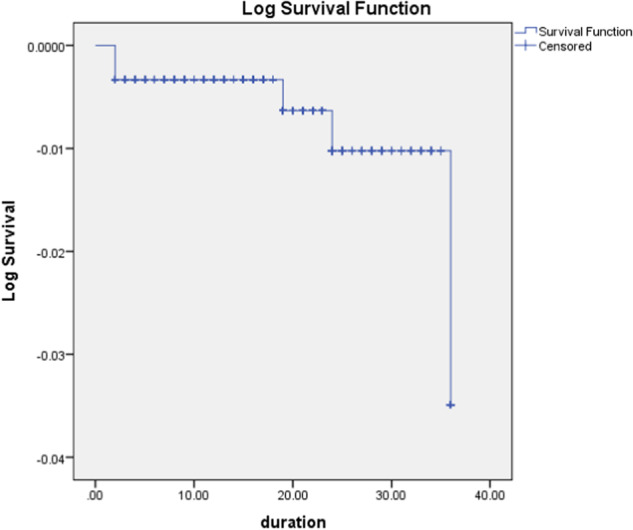
Kaplan–Meier calculations of survival from removal or revision at 1, 2, and 3 years.

Kaplan–Meier graphs show the number of patients with ED who survived reoperation divided by the number of patients with ED who were at risk for revision. Survival calculations were shown for 12 months, 24 months and for 36 months. Kaplan–Meier statistical survival curves were 98.8%, 98.9%, and 99.2%, respectively (Fig. 2).

## Discussion

The medical literature shows that MPPs have lower satisfaction rates than IPPs [6]. A study from Ralph’s group of 504 United Kingdom patients spanning 25 years showed IPP satisfaction at 86.2% and MPP at 75.1% [10]. Despite this finding, there are many clinical situations where the MPP is preferred by physicians and patients alike. Patients with limited manual dexterity [11] are better served with an MPP than having to inflate and deflate an IPP pump [4, 8]. Other patients desire simplicity and choose MPP [11]. A patient suffering from ED and voiding difficulty with a buried penis is best served by an MPP rather than IPP [12]. Use of the MPP as a replacement or space holder for patients with priapism or salvage surgery for implant infection are other indications where MPP is a superior choice [13]. Many OUS physicians find patients with diabetes, spinal cord injury and Peyronie’s disease better treated with MPP than IPP [14]. Despite a recent AUA News article entitling MPP as the “forgotten implant” [11], Khera et al.’s review article considered that despite the paucity of MPP used in the US, there was definitely “still a place for malleable penile prosthesis” [6]

MPPs have been available for 85 years with multiple brands available worldwide [6]. If a new MPP comes to market, the first questions in the mind of both patient and physician are: is the device as safe as present models available and does the new model have any advantage over existing brands? This is the first paper to evaluate the initial safety from revision, removal or replacement for any reason of ED patients implanted with the new Rigicon Rigi10® MPP. The main purpose of this study was to obtain multi-center safety data from the majority of the devices implanted worldwide since the inception in 2019 of the use of the Rigicon Rigi10® MPP as a treatment for ED. Our study design included virtually every first time Rigi10® performed since the device was first introduced. To adequately validate the safety of usage, we desired the various geographic and diverse forms of urological application, which spanned 15 countries.

Surgeries were performed by 46 high-volume, experienced implanters. Due to its recent introduction, Rigi10® implanted in the US were included, but the number was small (*n* = 64) with the first few implants (*n* = 15) lacking the hydrophilic coating for regulatory reasons.

MPPs were an improvement from the original semi-rigid rod prosthesis of the 1980s. The inclusion of a trimmable cylinder instead of multiple sizes made inventory more efficient and an imbedded wire made concealment for the patient much better. Present devices can bend, although limited, without causing changes in penile length but facilitating concealment. The best advantages of MPP are that their cost is low when compared to inflatable models, postoperative morbidity is less, and it is surgically quicker and easier to implant [6]. In this study, Rigi10™ MPP was successful in treating ED in 603 of 606 patients (99.5%). Only 3 patients were dissatisfied with the result and opted for IPP substitution. Rigi10®’s safety was demonstrated convincingly; according to our results Rigi10® MPP had no mechanical malfunction and the risk of prosthesis erosion (0.17%), infection (0.33%), or patient dissatisfaction (0.49%) was quite low. Less than 1% of all implants required reoperation for any reason.

Anecdotal remarks from the implanting physicians who have experience with a variety of competitive MPPs, indicated that compared to its competitors, the best features of Rigi10™ MPP were that the supple bending capacity of its rods simplifies implantation through a smaller corporotomy and the availability of extra widths of cylinders facilitates more accurate penile girth attainment. Support for this opinion comes from (Table 3) which indicates that the size 12 mm cylinder, which is unavailable in competitive devices, was the second most used cylinder width in this Rigi10 study.

The new Rigicon Rigi10™ was introduced in OUS in 2019 and in the US market in 2021. For the last 30 years, there have been only two manufacturers of MPPs in the US market [6]. These two companies have marketed a variety of MPPs over the last 50 years. American Medical Systems (now Boston Scientific) has produced the AMS 600, AMS 650, Duraphase, Dura II, Spectra and the current device called Tactra. Mentor’s (now Coloplast) first MPP was the Accuform®. When a hydrophilic coating was added, it was renamed Genesis®. For most of the first 20 years of this century, the only two MPPs available in the USA were the Spectra® and the Genesis®. The Spectra® was more expensive to manufacture as its superior bending mechanism was polysulfone segments strung on a wire. After Boston Scientific acquired AMS, they replaced the Spectra® with the Tactra® in 2019. The expensive polysulfone segments strung on a wire were replaced Nitinol®.

One of the benefits of MPP is a lesser need for revision surgery when compared to inflatable devices [6]. When we compare the new Rigicon MPP to the new Rigicon IPP, 2-year device survival from reoperation was 93.7% for the multicomponent inflatable compared to 99% for the malleable at 3 years [9]. Lacy et al. outlined a retrospective study of Veteran’s Hospital patients comparing IPP and MPP. Of 6586 patients over 13 years, 87% received IPP and 13% got MPP. The MPP had a better 1-, 5- and 10-year survival from revision [15].

MPP does not contain movable parts that may have mechanical problems or fluid-filled components which can leak. A study by Minervini et al. following 393 MPP for up to 25 years showed only a 0.5% mechanical malfunction rate [10]. In our study, the mean follow-up period was 21.6 months and there were no mechanical malfunctions; Kaplan–Meier’s analysis revealed cumulative survival of the Rigi10® at 12, 24, and 36 months of 98.8%, 98.9%, and 99.2%. Undoubtedly, this study is limited by the fact that data to obtain the safety profile was obtained from voluntarily filled out and mailed PIFs. While the motivation to fill out these forms is quite high to achieve the device’s lifetime guarantee, it stands to reason that occasional implanted patients were missing from the accumulated number for the study. For this same reason, an occasional revision operation could have been performed in another hospital by a surgeon different from the original implanter, and the revision or removal surgeon was not motivated to document the original PIF. A belief that this variance occurs can be tempered by the fact that -these operations result in a contaminated prosthesis. At all hospitals, it is the requirement that any explanted medical device must be submitted to pathology and then subsequently returned to the manufacturer. Upon receipt of the explantated device, the manufacturer authenticates the original PIF. Because IPP is an infrequently performed surgery and developing meaningful outcome data requires many patients from many sources, PIF data have been trusted for many seminal penile prosthesis papers in our prosthetic urology literature [7, 8].

## Conclusions

The new malleable prosthesis, Rigi10® showed remarkable early safety from the necessity of reoperation in a multi-center study of the initial patients receiving the implant. Followed up to 3 years with a mean of 21.6 months, the Rigicon Rigi10™ had no mechanical breakage and only required revision/removal surgery in less than 1% of 605 patients.

## Data Availability

The datasets generated during and/or analyzed during the current study are available from the corresponding author upon reasonable request.
